# Supplementary material for: Knowledge, attitudes and practices (KAP) on COVID-19 among the general population in most affected districts of Nepal
Source: PLOS Glob Public Health. 2023 Jul 28;3(7):e0001977. doi: 10.1371/journal.pgph.0001977 (PMC10381065; doi:10.1371/journal.pgph.0001977)
Supplement: S1 Questionnaire — (DOCX) [file pgph.0001977.s001.docx]

**Questionnaire**

Sample ID: ………………………………………………

Name: ………………………………………………………..

Address:…………………….………Province……………………..……District ………………………………Municipality

Contact no…………………………………………….

**A. Personal information**

**Q.N.1** What is your gender?

i) Male ii) Female

**Q.N.2** What is your marital status?

i) Married ii) Unmarried

**Q.N.3** Please mention about the major occupation.

i) Agriculture ii) Service iii) Business iv) Labor v) House rent vi) Self-employed vii) Remittance viii) Others ………………………

**Q.N.4** What is your age?

i) <20 years ii) 20-30 years iii) 31-40 years iv) 41-50 years v) >50 years

**Q.N.5** What is your highest education?

I) No Education ii) Literate iii) Basic Education iv) Secondary Education v) Undergraduate vi) Graduate & above

**Q.N.6** Please mention about the monthly income.

i) <NPR 5000 ii) NPR 5000-10,000 iii) NPR 10,000-15,000 iv) NPR 15,000-20,000 v) NPR >20,000

**B. Knowledge, Attitudes and Practices (KAP)**

***Knowledge***

**Q.N.7** Are you familiar about the following symptoms of COVID-19? **Please choose the appropriate answers. (√)**

| **S.N.** | **Symptoms** | **Yes** | **No** |
| --- | --- | --- | --- |
| 1. | Fever |  |  |
| 2. | Dry cough |  |  |
| 3. | Tiredness |  |  |
| 4. | Aches and pains |  |  |
| 5. | Sore throat |  |  |
| 6. | Diarrhoea |  |  |
| 7. | Conjunctivitis |  |  |
| 8. | Headache |  |  |
| 9. | Loss of taste or smell |  |  |
| 10. | A rash on skin, or discoloration of fingers or toes |  |  |
| 11. | Difficulty breathing or shortness of breath |  |  |
| 12. | Chest pain or pressure |  |  |
| 13. | Loss of speech or movement |  |  |

**Q.N.8** Do you know about the following preventive measures to be protected from COVID-19? **Please choose the appropriate answers. (√)**

| **S.N.** | **Items** | **Yes** | **No** |
| --- | --- | --- | --- |
| 1. | Washing hand with soap for 20 seconds or use alcohol-based sanitizer |  |  |
| 3. | Cover mouth and nose while coughing and sneezing |  |  |
| 4. | Maintain social distance up to 6ft |  |  |
| 5. | Use a mask while going outside |  |  |
| 6. | Avoid crowd |  |  |
| 7. | Stay at home for safety |  |  |

**Q.N.9** Do you know about the pre-condition to stay in quarantine? **Please choose the appropriate answers. (√)**

| **S.N.** | **Items** | **Yes** | **No** |
| --- | --- | --- | --- |
| 1. | Stayed with infected person within 6 ft. of distance for 15 minutes or more |  |  |
| 2. | Take care of infected person within house |  |  |
| 3. | Touching the infected person |  |  |
| 4. | Using the Utensils of infected person |  |  |
| 5. | Contact with droplets while sneezing and coughing by infected person |  |  |

**Q.N.10** Do you know proper diet and exercise will help to boost your immune system?

i) Yes ii) No

**Q.N.11** Do you know that SARS-CoV-2 virus can transfer from aerosols?

i) Yes ii) No

**Q.N.12** Do you know, SARS- CoV-2 virus can survive 72 hours on plastic and stainless steel?

i) Yes ii) No

**Q.N.13** Do you know, SARS- CoV-2 can survive 24 hours on cardboard?

i) Yes ii) No

**Q.N.14** Do you know, SARS- CoV-2 will survive 4 hours on copper?

i) Yes ii) No

**Q.N.15** Do you know, SARS- CoV-2 will survive 3 hours in aerosols?

i) Yes ii) No

**Q.N.16** Do you know mobile phone surface are potential carrier of corona virus that frequently comes in contact with nose and mouth increase the risk of infection?

i) Yes ii) No

**Q.N.17** Do you know about new variants of SARS-CoV-2?

i) Yes ii) No

**Q.N.18** Do you know that co-morbidities of following chronic diseases among the COVID-19 infected persons increase the death rate? **Please choose the appropriate answers. (√)**

| **S.N.** | **Items** | **Yes** | **No** |
| --- | --- | --- | --- |
| 1. | Hypertension |  |  |
| 3. | Diabetics |  |  |
| 4. | Cardiovascular disease |  |  |
| 5. | Kidney disease |  |  |
| 6. | Liver disease |  |  |
| 7. | Cerebrovascular disease |  |  |
| 8. | Cancer |  |  |
| 9. | Lung disease |  |  |

***Attitudes***

**Q.N.19** **Please choose the appropriate answers. (√)**

| **S.N.** | **Items** | **Strongly agree** | **Agree** | **Neutral** | **Disagree** | **Strongly disagree** |
| --- | --- | --- | --- | --- | --- | --- |
| 1. | I am willing to adhere the all the preventive measures to reduce the risk of transmission. |  |  |  |  |  |
| 2. | I believe staying in quarantine if come in contact with infected person will reduce the transmission. |  |  |  |  |  |
| 3. | I think keeping myself safe is crucial in breaking the chain of transmission of COVID-19. |  |  |  |  |  |
| 4. | I believe that exercise and diet will increase immunity to fight against the COVID-19. |  |  |  |  |  |
| 5. | I am anxious that it will lead to a difficult situation with further loss of life and property if failed to take careful steps on time. |  |  |  |  |  |
| 6. | I believe the pandemic will end soon. |  |  |  |  |  |

***Practices***

**Q.N.20** How often do you practice following measures during lockdown? **Please choose the appropriate answers. (√)**

| **S.N.** | **Items** | **Always** | **Most of the time** | **Sometime** | **Rarely** | **Never** |
| --- | --- | --- | --- | --- | --- | --- |
| 1. | Washing hands with soap for 20 sec or use alcohol-based sanitizer |  |  |  |  |  |
| 2. | Cover mouth and nose while coughing and sneezing. |  |  |  |  |  |
| 3. | Maintain social distance up to 6ft |  |  |  |  |  |
| 4. | Use a mask while going outside |  |  |  |  |  |
| 5. | Avoid crowd |  |  |  |  |  |
| 6. | Stay at home for safety |  |  |  |  |  |
| 7. | Clean mobile phone with sanitizer after coming from outside |  |  |  |  |  |
| 8. | Having proper diet |  |  |  |  |  |
| 9. | Doing regular exercise |  |  |  |  |  |

**Q.N.21** How often do you practice following measures after lockdown? **Please choose the appropriate answers. (√)**

| **S.N.** | **Items** | **Always** | **Most of the time** | **Sometime** | **Rarely** | **Never** |
| --- | --- | --- | --- | --- | --- | --- |
| 1. | Washing hands with soap for 20 sec or use alcohol-based sanitizer |  |  |  |  |  |
| 2. | Cover mouth and nose while coughing and sneezing. |  |  |  |  |  |
| 3. | Maintain social distance up to 6ft |  |  |  |  |  |
| 4. | Use a mask while going outside |  |  |  |  |  |
| 5. | Avoid crowd |  |  |  |  |  |
| 6. | Stay at home for safety |  |  |  |  |  |
| 7. | Clean mobile phone with sanitizer after coming from outside |  |  |  |  |  |
| 8. | Having proper diet |  |  |  |  |  |
| 9. | Doing regular exercise |  |  |  |  |  |
