# Supplementary material for: Knowledge, attitudes and practices (KAP) on COVID-19 among the general population in most affected districts of Nepal
Source: PLOS Glob Public Health. 2023 Jul 28;3(7):e0001977. doi: 10.1371/journal.pgph.0001977 (PMC10381065; doi:10.1371/journal.pgph.0001977)
Supplement: Attachment — Submitted filename: Response to comments_PLOS Global_27th Nov.docx [file pgph.0001977.s003.docx]

**Response to comments of Reviewer 1**

**The title selection is fair enough to analyze the behaviour of people, it is need of the hour in such case it becomes very important to have set of variables which are not overlapping or common amongst all. It is also important to mention the time of data collection, which is missing.**

***Response***

\# *We have selected the variables which are very important to be considered and common amongst all according to our preliminary observation.*

*# We added following information regarding the time of data collection.*

*Line 126*

*# The data was collected from 21^st^ March 2021 to 12^th^ April 2021.*

**Sample size should be scientifically calculated. Research Methodology section is weak.**

***Response***

*We have added the information on sample size calculation*

*Line 101 to111*

*The sample size (n) was determined by following the formula given by Arkin and Colton (1963) (45).*

$$n=\frac{N*z^{2}*P (1-P)}{N*d^{2}+z^{2}*P (1-P)}$$

*Where,*

*n = sample size*

*N = Total population size (total population of eight selected districts) = 61, 98,158 (46).*

*Z = confidence interval (at 95% level z =1.96)*

*P = estimated population proportion (0.5)*

*d = error limit of 5% (0.05)*

*The minimum sample size required for the study was 400.*

*A convenient non-probability sampling method was considered due to the inappropriateness of the probability sampling during the period of the pandemic. The total sample collected was 702*

*# We have significantly improved our methodology section as per suggestion. Following changes were made*

*Line 133 to 163 (Measurements)*

*Questions related to knowledge of symptoms, preventive measures, pre-condition to stay in quarantine if come into contact with infected person, increase in risk of death due to co-morbidities among the COVID-19 infected person, diet and exercise to boost immune system, transmission of SARS-CoV-2 through aerosols, life duration of SARS-CoV-2 in fomites (plastic and stainless steel, cardboard, copper) and aerosols, high risk of transmission through mobile phone and new variants of SARS-CoV-2 were included in the questionnaire. Thirteen binary questions (yes/ no) questions were used to assess the knowledge of ‘symptoms’ with a total score range of 0-13. The knowledge of symptoms was dichotomized with a cutoff value of 10 as those having good knowledge (>10) and poor knowledge (≤10). There were six binary questions related to knowledge on ‘preventive measures’ with total score in the range 0-6. The knowledge on preventive measures was dichotomized with cutoff value of 6 as having good and poor knowledge. Similarly, five binary questions related to knowledge on ‘precondition to stay in quarantine if come into contact with infected person’ with the total score in the range of 0-5 were categorized as having good and poor knowledge with a cutoff value of 5. The knowledge level on increase in risk of death due to co-morbidities among the COVID-19 infected person includes eight diseases. The score on knowledge on increase in risk of death due to co-morbidities range from 0-8 and was dichotomized with cutoff value of 6 as having good knowledge (>6) and poor knowledge (≤6). The questions regarding the knowledge on diet and exercise to boost immune system, transmission of SARS-CoV-2 through aerosols, life duration of SARS-CoV-2 in fomites (plastic and stainless steel, cardboard, copper) and aerosols, high risk of transmission through mobile phone, new variants of SARS-CoV-2 were simply responded as ‘yes’ or ‘no’. Lastly, all these twelve final items were used to measure the overall knowledge level of the general population with total score ranging from 0-12 with overall good (>7) and poor knowledge (≤7) on COVID-19 with a cutoff value of 7. The median values were calculated to determine the cut off levels.*

*Attitude level was measured using a five-point Likert Scale questions where the responses varied from (1) strongly disagree to (5) strongly agree. The total score of the attitude questionnaire was the sum of scores of all the items ranging from 6 to 30 which was categorized into good (>26) and a bad attitude (≤26) with a cutoff value of 26 (median). Practice level after lockdown was measured using a five-point Likert Scale questionnaire for prevention of COVID-19 from (1) never to (5) always. The total score of practice questionnaires ranging from 9 to 45 was categorized as good practice (>33) and poor practice (≤33) with a cut-off value of 33 (median).*

*Line 174 to 184 (Statistical Analysis)*

*Chi-square test was used to evaluate the differences in knowledge, attitude and practice in relation to socio-demographic characteristics.. The normality of data was checked using Shapiro-Wilk test (p < 0.05 showing non-normal distribution of continuous variable). Therefore, the Wilcoxon Rank Test was used to assess the differences in practice for prevention before and after lockdown amng the respondents.*

*Line 182 to 183*

*The significance level was set at p < 0.05 for all the statistical analysis.*

**Findings must have a unique outcome of this study which is lacking. No extra-ordinary solution or suggestive measures are produced in the conclusion. The conclusion should be concise, author’s contribution in form of explaining the strategies and action plan or a practice which may be adopted can be proposed.**

***Response***

*# Though, there are several studies conducted on KAP on COVID-19, we have included the distinctive information that most of the study didn’t cover such as, knowledge on increase in risk of death due to co-morbidities, mode of transmission including fomites, the overall symptoms mentioned by WHO, detail prevention practice comprising variation in behavior during and after lockdown etc. in a single study. In context of Nepal also there were limited study conducted regarding KAP on COVID-19 and lacks the consideration of following important variables. Therefore, the study findings are imperative.*

*# We have revised the conclusion as suggested.*

*Line 405 to 419 (Conclusion)*

*Findings from our study are imperative to understand the perceived knowledge, attitude and behavior among the general population during the COVID-19 pandemic.*

*The people residing in urban areas, with white-collar occupations, high-income, and high-education were good at KAP on COVID-19. In addition, females seem to be good at practicing preventive behavior although their knowledge level was lower compared to male.*

*The variation in KAP on COVID-19 among the people with varying socio-economic conditions was due to the difference in the level of their understanding and available resources. The study result recommends the subsequent improvement in existing plans, policies, guidelines with targeted approach and their effective implementation including accessibility of resources among all the population for the compliance of preventive measures, development of better communication strategy for their effective implementation considering effectual dissemination of information as well as infodemic management. The multifaceted approach with the significant contribution of concerned stakeholders and their proper co-ordination including government bodies is essential to deal with the challenges during such a health crisis with effective utilization of the available limited resources.*

**The future scope, implications and limitations of the study is missing which can be added to enhance manuscript value.**

***Response***

*# We have added the details in the manuscript as per suggestions*

*Line 398 to 404*

*There are some limitations of the study. It is cross-sectional study. So it cannot provide the evidence over a period of time. The non-probability sampling method was used due to the unsuitability in conducting probability sampling during the health crisis. The implications of the study is the findings provides timely understanding among the policy makers for revising the existing policies and recognizing and emphasizing the target population to reduce the severity of the pandemic. The relationship of culture, social norms, ethnicity and information behavior with KAP on COVID-19 among the general population was not included in this study. The future study considering these factors is crucial for detail understanding.*

*# We have further elaborated the discussion section with additional implications of the study*

*Line 370 to 377*

*The monitoring of consistency of information shared through different media is also important to avoid the confusion (69). Therefore, our study result emphasized the need of establishment of risk communication protocol* *with clear definition of tasks among the government bodies and concerned authorities. It is indispensable to understand the people’s adjustment towards recommendation provided to be safe against COVID-19. For this, the establishment of two way communication platforms and community feedback mechanisms are very helpful to understand and engage communities in discussion and communication. The community health volunteers should be mobilized for door to door visit to aware the people.*

**References are sufficient and appropriate keeping in view that it is a chapter. But yes, few recent studies can be included. I would recommend that more relevant literature can be added under the heading of 'Literature Review' to intensify the thought process in the reader of the manuscript.**

***Response***

*# We have added the additional relevant literature in the reference section as follows.*

*Line 526 to 648 (Reference)*

*27. Chisty, M. A., Islam, M. A., Munia, A. T., Rahman, M. M., Rahman, N. N., & Mohima, M. (2021). Risk perception and information-seeking behavior during emergency: An exploratory study on COVID-19 pandemic in Bangladesh. International Journal of Disaster Risk Reduction, 65, 102580.*

*28. Commodari, E. (2017). The role of sociodemographic and psychological variables on risk perception of the flu. Sage Open, 7(3), 2158244017718890.*

*35. Getawa S, Aynalem M, Bayleyegn B, Adane T (2022) Knowledge, attitude and practice towards COVID-19among secondary school students in Gondar town, Northwest Ethiopia. PLoS ONE 17(5):e0268084.https://doi.org/ 10.1371/journal.pone.0268084*

*45. Arkin H, and Colton R. Tables for Statisticians. 1963. New York: Barnes and Noble.*

*46. Central Bureau of Statistics, Nepal. National Population and Housing Census 2011*

*48. Malik, J. A., Ahmed, S., Shinde, M., Al-Marmash, M. H. S., Alghamdi, S., Hussain, A., & Anwar, S. (2022). The impact of COVID-19 on the comorbidities: a review of recent updates for combating it. Saudi Journal of Biological Sciences.*

*49. Gupta, R., Singh, B., Gautam, A., Singh, G., Khan, M., & Kumar, V. (2022). SARS-CoV-2 infection related mortality and comorbidities in a dedicated COVID-19 facility: A record based analysis from Uttar Pradesh. Asian Journal of Medical Sciences, 13(3), 3-10.*

*50. Piret, J., & Boivin, G. (2021). Pandemics throughout history. Frontiers in microbiology, 11, 631736*

*51. Mackowiak, P. A. (2021). Prior pandemics. looking to the past for insight into the COVID-19 pandemic. Journal of Community Hospital Internal Medicine Perspectives, 11(2), 163-170.*

*52. Katella, K. (2022). Omicron, Delta, Alpha, and more: What to know about the coronavirus variants. Yale Medicine, 28th February, available at: https://www. yalemedicine. org/news/covid-19-variants-of-concern-omicron (accessed 8th March, 2022).*

*53. Policy Research Institute. Situation of COVID-19 pandemic, management experiences and learning in different countries.*

*55. Ghio, D., Lawes-Wickwar, S., Tang, M. Y., Epton, T., Howlett, N., Jenkinson, E., ... & Keyworth, C. (2021). What influences people’s responses to public health messages for managing risks and preventing infectious diseases? A rapid systematic review of the evidence and recommendations. BMJ open, 11(11), e048750.*

*59. Banu, B., Chowdhury, S. H., Akter, N., Islam, K. R., Hossain, S. M., & Amin, M. R. (2021). Preventive Behaviors to Mitigate COVID-19: Urban-Rural Disparities of Densely Populated Country like Bangladesh. Journal of Environmental Science and Public Health, 5, 433-450.*

*60. Callaghan, T., Lueck, J. A., Trujillo, K. L., & Ferdinand, A. O. (2021). Rural and urban differences in COVID‐19 prevention behaviors. The Journal of Rural Health, 37(2), 287-295.*

*62. Dev R, Raparelli V, Bacon SL, et al. Impact of biological sex and genderrelated factors on public engagement in protective health behaviours during the COVID-19 pandemic: crosssectional analyses from a global survey. BMJ Open 2022;12:e059673. doi:10.1136/ bmjopen-2021-059673*

*69. Hung, L., & Lin, M. (2022). Clear, consistent and credible messages are needed for promoting compliance with COVID-19 public health measures. Evidence-Based Nursing, 25(1), 22-22.*

**Response to the comments of reviewer 2**

The reviewed article aims to identify Nepal's knowledge, attitude, and practice during the COVID-19 pandemic. The subject matter discussed by the article's Authors is undoubtedly essential. In my opinion, the paper's argument is built on a proper theoretical basis and concepts. In the introduction, the Authors described the pandemic issues in the context of Nepal, indicating current and potential future difficulties connected with the new coronavirus. The economic situation of the country, as well as the fragile and insufficient health care system, justify undertaking the research. The authors invoked the previous studies conducted in other countries and emphasized the gap concerning the KAP study on COVID-19 in Nepal.

**In the materials and methods, Author/s described the technique, research area, research group, instrument, and research analysis process. The significant doubt is point 2.4 Measurement, where authors indicated the cutoff value referring to respondents' knowledge, attitude, and practice. Lack of information or references concerning the criterium according to which the cutoff value for each dimension was established.**

***Response***

*# We have added the details in methodology section as follows:*

*Line 133 to 163. (Measurements)*

*Questions related to knowledge of symptoms, preventive measures, pre-condition to stay in quarantine if come into contact with infected person, increase in risk of death due to co-morbidities among the COVID-19 infected person, diet and exercise to boost immune system, transmission of SARS-CoV-2 through aerosols, life duration of SARS-CoV-2 in fomites (plastic and stainless steel, cardboard, copper) and aerosols, high risk of transmission through mobile phone and new variants of SARS-CoV-2 were included in the questionnaire. Thirteen binary questions (yes/ no) questions were used to assess the knowledge of ‘symptoms’ with a total score range of 0-13. The knowledge of symptoms was dichotomized with a cutoff value of 10 as those having good knowledge (>10) and poor knowledge (≤10). There were six binary questions related to knowledge on ‘preventive measures’ with total score in the range 0-6. The knowledge on preventive measures was dichotomized with cutoff value of 6 as having good and poor knowledge. Similarly, five binary questions related to knowledge on ‘precondition to stay in quarantine if come into contact with infected person’ with the total score in the range of 0-5 were categorized as having good and poor knowledge with a cutoff value of 5. The knowledge level on increase in risk of death due to co-morbidities among the COVID-19 infected person includes eight diseases. The score on knowledge on increase in risk of death due to co-morbidities range from 0-8 and was dichotomized with cutoff value of 6 as having good knowledge (>6) and poor knowledge (≤6). The questions regarding the knowledge on diet and exercise to boost immune system, transmission of SARS-CoV-2 through aerosols, life duration of SARS-CoV-2 in fomites (plastic and stainless steel, cardboard, copper) and aerosols, high risk of transmission through mobile phone, new variants of SARS-CoV-2 were simply responded as ‘yes’ or ‘no’. Lastly, all these twelve final items were used to measure the overall knowledge level of the general population with total score ranging from 0-12 with overall good (>7) and poor knowledge (≤7) on COVID-19 with a cutoff value of 7. The median values were calculated to determine the cut off levels.*

*Attitude level was measured using a five-point Likert Scale questions where the responses varied from (1) strongly disagree to (5) strongly agree. The total score of the attitude questionnaire was the sum of scores of all the items ranging from 6 to 30 which was categorized into good (>26) and a bad attitude (≤26) with a cutoff value of 26 (median). Practice level after lockdown was measured using a five-point Likert Scale questionnaire for prevention of COVID-19 from (1) never to (5) always. The total score of practice questionnaires ranging from 9 to 45 was categorized as good practice (>33) and poor practice (≤33) with a cut-off value of 33 (median).*

**The form of questions concerning knowledge and the binary possibility of answers (yes/no) also raises considerable doubts (Table 1; line 189; S.N.: K1-K12). In my opinion, the established form of items/questions (e.g., "Do you have good/proper knowledge of…"; "Do you know that/about…"; etc.) do not state the level of knowledge of the respondents and to draw conclusions in this regard.**

***Response***

*# The detailed information was added regarding knowledge item accordingly for more clarity.*

*Line 133 to 156 (Methodology: Measurements)*

*Line 225 (Findings)*

*# We have rephrased the items in the table for better understanding (Table 1)*

**Considering the respondents' attitude toward COVID-19, presented in Table 2 (line 202), items: A1; A2; A5 are unclear and should be formulated more precisely. There are also some doubts regarding item A6 (Table 2) and the significance of the question (Do you believe we will soon get rid of the COVID-19 pandemic?) and gained answers.**

***Response***

*# We have made correction as per the suggestion. We have constructed A1, A2 and A5 more precisely and rephrased the item A6 with more clarity.*

*Line 233 (Findings)*

*The corrected items are mentioned in Table 2*

**There is also no explanation of the research finding regarding the indicated item in the Discussion (line 279).**

***Response***

*# We have added the explanation of the finding as follows in the discussion section*

*Line 311. To 317*

*However, they were doubtful that the pandemic will end soon. The history of previous pandemics revealed that the health crisis lasted for years with massive loss of life and property had created the fear of prolonging of COVID-19 pandemic (50, 51). Although, many researches are carried out and many information are explored regarding SARS CoV-2, the virus is changing constantly and there are still many unknown facts regarding the risk of spread of new variants of concern that lead to the uncertainties (52). Moreover, the lack of strong control mechanism during health emergencies in developing countries had further diminish the trust of people upon the government and responsible authorities (53)*

**Moreover, in my opinion, using the Likert scale to measure the respondents' attitudes toward COVID-19 would be clearer if items in Table 2 were formulated as sentences, not questions.**

***Response***

*Line 233. (Findings)*

*# The items of attitude towards COVID-19 were formulated as sentences as per the suggestion (table 2).*

**Figure 4: Knowledge of pre-condition to stay in quarantine among the respondents (lines 170-174) and presented items are unclear, which makes this part difficult to understand by the reader.**

***Response***

*# We have improved this section and corrected the captain of Figure 4*

*Line 206 to 207 (Findings)*

*Fig 4: Knowledge of pre-condition to stay in quarantine among the respondents if come into contact with infected person*

**The descriptive analysis of obtained research results is too detailed.**

***Response***

*# We have shortened the description of descriptive analysis*

*Line 187 to 224 (Findings)*

***3.1 Knowledge***

*The study revealed that, very few proportions of respondents had knowledge of the symptoms of conjunctivitis (n = 159, 22.9%) followed by skin rashes or discoloration of skin or toes (n = 172, 24.7%) diarrhea (n = 258, 37%), whereas, a higher proportion of respondents had knowledge on fever (n = 699, 99.7%), dry cough (n = 680, 97%) and tiredness (n = 634, 90.3%), (Figure 2). Most of the respondents had good knowledge regarding several preventive measures. Higher proportion of them had good knowledge on covering of nose and mouth while sneezing and coughing (n = 700, 99.7%), using mask (n = 698, 99.4%) and necessity of washing hands or use of alcohol-based sanitizer (n = 692, 98.6%), (Figure 3). Similarly, higher proportion of respondents had knowledge of pre-conditions to stay in quarantine if come into contact with infected person such as contact with droplets while sneezing and coughing (n = 675 96.4%) followed by taking care of an infected person within house (n = 649, 93%) and using utensils of infected person (n = 630, 90.4%) (Figure 4). Considering the knowledge of increase in risk of death due to* *comorbidities among COVID-19 patients , many of the respondents had knowledge of increased risk due to lung disease (n = 604, 86.8%), followed by cardiovascular symptoms (n = 511, 73%), while very few had knowledge of increased risk due to cerebrovascular disease (n = 292, 41.8%) (Figure 5).*

*Table 1 comprising the overall knowledge on COVID-19 among the respondents further revealed that a major proportion of them (n = 655, 93.3%) had good knowledge about overall preventive measures regarding COVID-19. However, very few respondents had good knowledge of symptoms (n = 221, 32%) and risk of comorbidities among the COVID-19 infected patient (n = 283, 40.7%). Similarly, (n = 466, 67.1%) of respondents had overall knowledge of the pre-condition of quarantine if come into contact with COVID-19 infected person. Concerning the knowledge of survival of SARS CoV-2 in fomites, (n = 375, 53.6%) knew about its survival duration on plastic and stainless steel, (n = 280, 40%) on cardboard, (n = 306, 43.7%) on copper, and (n = 352, 50.2%) on aerosols.*

*Line 227 to 232.*

***3.2 Attitude***

*Regarding attitude, a major proportion of respondents (n = 473, 67.4%) strongly agreed that keeping themselves safe was crucial for breaking the chain of transmission of COVID-19 and (n = 424, 61.2%) strongly agreed that exercise and diet would increase the immunity to fight against COVID-19. However, a very small proportion of respondents (n = 99, 14.3%) strongly agreed that the pandemic will end soon (Table 2).*

**The comparison of practices of coronavirus prevention during and after lockdown are presented in two separate tables (Table 3; line 211 and Table 4; line 2018). Showing the differences in that matter in one table would make them more visible and clear.**

***Response***

*Line 241 (Findings)*

*We have merged the results of two tables into a single one (table 3)*

**Response to the comments of academic editor**

**While the manuscript is valuable and addressing an interesting and important topic, the research lacks clarity on some parts of methodology and the analysis of data. Use of parametric and non-parametric methods are not justified. The manuscript fails to present the required assumptions of normality.**

***Response***

*# The description in the manuscript was elaborated as per the suggestions*

*Line 175 to 179 (Methodology)*

*Chi-square test was used to evaluate the differences in knowledge, attitude and practice in relation to socio-demographic characteristics. The normality of data was checked using Shapiro-Wilk test (p < 0.05 showing non-normal distribution of continuous variable). Therefore, the Wilcoxon Rank Test was used to assess the differences in practice for prevention before and after lockdown among the respondents.*

*Line 182 to 183*

*The significance level was set at p < 0.05 for all the statistical analysis.*

**Further, the weak literature survey and lack of formation of hypothesis in the study provides little implications.**

***Response***

*# We have added the following details on the formulation of hypothesis.*

*Line 89 to 90 (Introduction)*

*In our study, we hypothesized the variation in knowledge, attitude and practice with difference in socio-demographic characteristics and also variation in preventive practice during and after lockdown.*

*# Following additional relevant literature in the reference section was added.*

*Line 526 to 648 (References)*

*27. Chisty, M. A., Islam, M. A., Munia, A. T., Rahman, M. M., Rahman, N. N., & Mohima, M. (2021). Risk perception and information-seeking behavior during emergency: An exploratory study on COVID-19 pandemic in Bangladesh. International Journal of Disaster Risk Reduction, 65, 102580.*

*28. Commodari, E. (2017). The role of sociodemographic and psychological variables on risk perception of the flu. Sage Open, 7(3), 2158244017718890.*

*35. Getawa S, Aynalem M, Bayleyegn B, Adane T (2022) Knowledge, attitude and practice towards COVID-19among secondary school students in Gondar town, Northwest Ethiopia. PLoS ONE 17(5):e0268084.https://doi.org/ 10.1371/journal.pone.0268084*

*45. Arkin H, and Colton R. Tables for Statisticians. 1963. New York: Barnes and Noble.*

*46. Central Bureau of Statistics, Nepal. National Population and Housing Census 2011*

*48. Malik, J. A., Ahmed, S., Shinde, M., Al-Marmash, M. H. S., Alghamdi, S., Hussain, A., & Anwar, S. (2022). The impact of COVID-19 on the comorbidities: a review of recent updates for combating it. Saudi Journal of Biological Sciences.*

*49. Gupta, R., Singh, B., Gautam, A., Singh, G., Khan, M., & Kumar, V. (2022). SARS-CoV-2 infection related mortality and comorbidities in a dedicated COVID-19 facility: A record based analysis from Uttar Pradesh. Asian Journal of Medical Sciences, 13(3), 3-10.*

*50. Piret, J., & Boivin, G. (2021). Pandemics throughout history. Frontiers in microbiology, 11, 631736*

*51. Mackowiak, P. A. (2021). Prior pandemics. looking to the past for insight into the COVID-19 pandemic. Journal of Community Hospital Internal Medicine Perspectives, 11(2), 163-170.*

*52. Katella, K. (2022). Omicron, Delta, Alpha, and more: What to know about the coronavirus variants. Yale Medicine, 28th February, available at: https://www. yalemedicine. org/news/covid-19-variants-of-concern-omicron (accessed 8th March, 2022).*

*53. Policy Research Institute. Situation of COVID-19 pandemic, management experiences and learning in different countries.*

*55. Ghio, D., Lawes-Wickwar, S., Tang, M. Y., Epton, T., Howlett, N., Jenkinson, E., ... & Keyworth, C. (2021). What influences people’s responses to public health messages for managing risks and preventing infectious diseases? A rapid systematic review of the evidence and recommendations. BMJ open, 11(11), e048750.*

*59. Banu, B., Chowdhury, S. H., Akter, N., Islam, K. R., Hossain, S. M., & Amin, M. R. (2021). Preventive Behaviors to Mitigate COVID-19: Urban-Rural Disparities of Densely Populated Country like Bangladesh. Journal of Environmental Science and Public Health, 5, 433-450.*

*60. Callaghan, T., Lueck, J. A., Trujillo, K. L., & Ferdinand, A. O. (2021). Rural and urban differences in COVID‐19 prevention behaviors. The Journal of Rural Health, 37(2), 287-295.*

*62. Dev R, Raparelli V, Bacon SL, et al. Impact of biological sex and genderrelated factors on public engagement in protective health behaviours during the COVID-19 pandemic: crosssectional analyses from a global survey. BMJ Open 2022;12:e059673. doi:10.1136/ bmjopen-2021-059673*

*69. Hung, L., & Lin, M. (2022). Clear, consistent and credible messages are needed for promoting compliance with COVID-19 public health measures. Evidence-Based Nursing, 25(1), 22-22.*
