# Supplementary material for: Knowledge, attitudes and practices (KAP) on COVID-19 among the general population in most affected districts of Nepal
Source: PLOS Glob Public Health. 2023 Jul 28;3(7):e0001977. doi: 10.1371/journal.pgph.0001977 (PMC10381065; doi:10.1371/journal.pgph.0001977)
Supplement: Attachment — Submitted filename: Response to Editor.docx [file pgph.0001977.s004.docx]

5 May, 2023

Academic Editor

PLOS Global Public Health

Dear Sir/Madam,

Thank you for your valued suggestion. We wish to share the revised version of our manuscript entitled “Knowledge, attitudes and practices (KAP) on COVID-19 among the general population in most affected districts of Nepal” submitted in ***PLOS Global Public Health***.

We included the revised figure file, revised manuscript without track change and with track change mode incorporating the suggestion. The response is mentioned below for your reference.

**Journal Requirements:**

**1. Fig 1: please (a) provide a direct link to the base layer of the map (i.e., the country or region border shape) and ensure this is also included in the figure legend; and (b) provide a link to the terms of use / license information for the base layer image or shapefile. We cannot publish proprietary or copyrighted maps (e.g. Google Maps, Mapquest) and the terms of use for your map base layer must be compatible with our CC-BY 4.0 license.**

**Note: if you created the map in a software program like R or ArcGIS, please locate and indicate the source of the basemap shape file onto which data has been plotted.**

**If your map was obtained from a copyrighted source please amend the figure so that the base map used is from an openly available source. Alternatively, please provide explicit written permission from the copyright holder granting you the right to publish the material under our CC-BY 4.0 license.**

**Please note that the following CC BY licenses are compatible with PLOS license: CC BY 4.0, CC BY 2.0 and CC BY 3.0, meanwhile such licenses as CC BY-ND 3.0 and others are not compatible due to additional restrictions.**

***Response***

*We have made a revision in the figure file as follows and revised the caption in the manuscript accordingly.*

*Fig 1: Location of the selected districts (Bhaktpur, Chitwan, Kaski, Kathmandu, Lalitpur, Morang, Rupandehi, and Sunsari)*

*The darkest grey thick line covering the grey area represents the country boundary of Nepal and the soft grey lines inside the country boundary represent the administrative boundaries of Nepal separating different districts. The location of selected districts for the study is presented by rectangles. Country boundary and administrative boundaries shape files of Nepal retrieved from the Database of Open Data Nepal website (*[*https://opendatanepal.com/dataset/new-political-and-administrative-boundaries-shapefile-of-nepal*](https://opendatanepal.com/dataset/new-political-and-administrative-boundaries-shapefile-of-nepal)*), under an open license (CC BY 4.0):* [*https://opendatanepal.com/*](https://opendatanepal.com/)*. Figure created with QGIS software version 3.28, an open source Geographic Information System (GIS) licensed under the GNU General Public License (*[*https://bit.ly/2BSPB2F*](https://bit.ly/2BSPB2F))

Thank you very much for your consideration.

Sincerely,

Rabin Malla, PhD

Executive Director

Center of Research for Environment, Energy and Water
